# Subclinical Hypoventilation in Dogs Undergoing Ventral Slot Decompressive Surgery for Cervical Myelopathy Due to Intervertebral Disc Herniation

**DOI:** 10.3389/fvets.2021.777052

**Published:** 2021-11-04

**Authors:** Melissa N. Andruzzi, Bradley T. Simon, Elizabeth Boudreau

**Affiliations:** Department of Small Animal Clinical Sciences, Texas A&M University, College Station, TX, United States

**Keywords:** blood gas, hypercapnia, perioperative, sedation, respiratory

## Abstract

The objective of this prospective cohort study was to document the occurrence of post-operative hypoventilation in dogs undergoing decompressive ventral slot or hemilaminectomy for the treatment of intervertebral disc herniation (IVDH). Twenty dogs undergoing ventral slot surgery and 20 dogs undergoing hemilaminectomy surgery for the treatment of IVDH that presented to XX between 2017 and 2020 were enrolled. Dogs were anesthetized using a standard protocol. Blood gas samples were taken at up to 11 time points beginning during anesthetic recovery and continuing for a maximum of 72 h post-operatively. Dogs with cervical lesions that were non-ambulatory before surgery had more evidence of subclinical hypoventilation in the immediate peri-extubation period than dogs with less severe injuries or those undergoing hemilaminectomy surgery. We found no difference in the ventilation status in dogs undergoing cervical or thoracolumbar decompressive surgery for IVDH from 8 to 72 h post-operatively. Other markers of acid-base status indicated that subclinical hypoventilation within the peri-extubation period was transient and self-limiting. There was a moderate positive correlation between sedation scores and estimated PaCO_2_. These data suggest that dogs with severe cervical spinal cord injuries may be at risk for subclinical hypoventilation in the immediate peri-extubation period. Increased sedation may be correlated with decreased ventilatory status in dogs recovering from decompressive vertebral column surgery.

## Introduction

Cervical myelopathies are associated with many perioperative complications attributed to the anatomic intimacy between the cervical spinal cord and vital structures, such as cardiovascular and respiratory tracts. Such complications include cardiac dysfunction and arrest, hypotension, aspiration pneumonia, hypoventilation, and seizure activity (1–8). Risk for clinical hypoventilation associated with cervical surgery is multifactorial. Dogs with cervical lesions are more likely to undergo anesthesia more than once, may have more difficulty clearing bronchial secretions, and may have impaired innervation to the diaphragm and intercostal muscles (4, 9).

Risk of respiratory complications is often compared between patients with cervical spinal injuries and patients with spinal injuries in more caudal locations (1, 6). Java et al. (4) reported that dogs that underwent cervical decompressive surgeries were six times as likely to develop post-operative pneumonia than post-operative patients with more caudal lesions. Despite this, the incidence of post-operative aspiration pneumonia following the most common surgical approach to the canine cervical region, the ventral slot, was reported in a large retrospective study to be just 0.37% (8).

There is variability in the reported association of clinical hypoventilation in animals with cervical spinal cord lesions. Beal et al. (1) found that 4.9% of dogs undergoing surgery due to cervical spinal disorders developed life-threatening hypoventilation that required positive-pressure ventilation (PPV). In a more recent study that examined 129 dogs anesthetized for diagnosis or treatment of intervertebral disc disease (IVDD) in the cervical region, only one dog developed hypoventilation severe enough to necessitate PPV (4). Additionally, in a larger and more targeted retrospective study, 546 dogs underwent ventral slot decompressive surgery for cervical IVDD, and only one required post-operative mechanical ventilation for the treatment of neurogenic hypoventilation (8).

While severe events of hypoventilation necessitating PPV have been reported on numerous occasions in association with cervical myelopathies and are easy to identify in a clinical setting, it is unknown how often subclinical hypoventilation may occur in this same patient group and may contribute to morbidity and mortality. Therefore, the primary objective of this study was to document the occurrence of subclinical hypoventilation in dogs undergoing a ventral slot for the treatment of intervertebral disc herniation (IVDH) compared to dogs undergoing hemilaminectomies for the treatment of thoracolumbar IVDH.

## Materials and Methods

This study was approved by the Institutional Animal Care and Use Committee of XX (AUP 2017-0238 CA) and all dogs were enrolled with informed owner consent. The manuscript is reported according to the Animal Research: Repeating of *in vivo* Experiments (ARRIVE 2.0) guidelines (10).

### Animals

In the present study, 40 client-owned dogs who were presented to the XX clinic between November 2017 and May 2020 were enrolled based on the following inclusion criteria: at least 1 year of age, at least 3 kg in body weight, presented for pain or neurologic dysfunction attributable to a cervical or thoracolumbar myelopathy that was ultimately determined to be due to IVDH by magnetic resonance imaging (MRI), and underwent decompressive surgery for the disc herniation under the same anesthetic event as the MRI. Dogs were under anesthesia at the time of enrollment, so ventilatory status could not be assessed at the time of enrollment. Dogs that exhibited clinical hypoventilation (as defined below) during the data collection period would have been removed from the study, but no enrolled dog met that criterion.

Dogs were separated into two groups: One group comprised 20 dogs with a cervical IVDH who underwent a ventral slot and the other comprised 20 dogs with a thoracolumbar IVDH who underwent a hemilaminectomy.

### Localization and Severity of Spinal Cord Injury

By convention, thoracic spinal cord segments are abbreviated as T, lumbar segments as L, and cervical segments as C in this report. Dogs with a compressive disc herniation affecting the T3–L3 spinal cord segments were classified as having thoracolumbar lesions. Dogs with compressive disc herniation affecting the C1–T2 spinal cord segments were classified as having cervical lesions.

We defined the severity of spinal cord injury, determined pre-operatively, according to the Modified Frankel Score (MFS) (11). For evaluation of ventilation as a function of severity of spinal cord injury, dogs were stratified into those that had clinical signs of spinal cord dysfunction severe enough to be classified as non-ambulatory (dogs with MFS scores of 3 or less) and those that did not (dogs with MFS scores of 4 or 5).

### Anesthesia and Perioperative Protocol

All dogs had pre-anesthetic physical and neurologic exams as well as pre-anesthetic diagnostics (including, but not limited to a complete blood count, serum biochemistry, venous blood gas analysis, and thoracic radiographs). Dogs then underwent general anesthesia using a standardized perioperative protocol for an MRI of the spinal cord and decompressive surgery for IVDH.

All dogs were pre-medicated with 0.2 mg/kg of methadone intravenously (IV) through a previously placed cephalic or saphenous IV catheter. Propofol was administered IV up to 4 mg/kg and to effect until endotracheal intubation was achievable with an appropriately sized cuffed endotracheal tube. Endotracheal tubes were attached to a rebreathing circuit and dogs were administered Sevoflurane (Akorn, Lake Forest, IL, USA) mixed in a carrier gas of 100% oxygen.

Heart rate (HR), end-tidal CO_2_ (EtCO_2_), respiratory rate (RR), end-tidal sevoflurane concentration (EtSevo), rectal or esophageal temperature, pulse arterial oxygen saturation (SpO_2_), and oscillometric blood pressure were recorded every 5 min using a multiparameter anesthesia monitor (Mindray, Mahwah, NJ, USA). All dogs were placed on a volume-controlled mechanical ventilator throughout the procedure and provided positive pressure ventilation to maintain an EtCO_2_ between 35 and 45 mmHg. No dog was recorded as having been given positive end-expiratory pressure (PEEP) during anesthesia.

Following induction and placement of monitoring devices, dogs were administered a fentanyl (Hospira, Lake Forest, IL, USA) IV bolus up to 5 mcg/kg and then started on a fentanyl constant rate infusion (CRI) (5–10 mcg/kg/h) using a syringe pump (B Braun Medical, Bethlehem, PA, USA). Fentanyl dose was adjusted intraoperatively based on fluctuations in physiological parameters (±20% change) and anesthetic depth. If fentanyl was deemed inadequate by the attending anesthesiologist a ketamine bolus (0.5–1 mg/kg) followed by a ketamine CRI (0.2–0.5 mg/kg/h) was administered.

Patients were aseptically prepped, and each underwent the scheduled surgical procedure. Anesthetic depth was monitored throughout and deemed appropriate as loss of palpebral reflex, ventromedial rotation of the eye, lack of jaw tone, and no physically manifest or substantial (±20% change) physiological response to surgical stimulation. Patients were discontinued from mechanical ventilation upon completion of the surgical procedure and allowed to spontaneously ventilate. If prolonged periods of apnea were identified by the attending anesthetists (e.g., >30–45 s without a noticeable breath) a small manual breath using positive pressure ventilation of 10–20 cm H_2_O peak inspiratory pressure was initiated. This was continued until the patient showed successive efforts to breathe spontaneously.

Post-operatively, for the first 24 h, a fentanyl CRI (3–4 mcg/kg/h) and a ketamine CRI (0.1–0.2 mg/kg/h), if needed, were used for pain management. Dosing adjustments and/or the need for ketamine were based on post-operative pain assessments performed at specific time points following surgery. At 24 h, dogs started a 4- to 6-h tapered off dosing of fentanyl ± ketamine, and then were transitioned to oral analgesics, consisting of either gabapentin (15–20 mg/kg PO q8 h) or pre-gabalin (2–4 mg/kg PO q8 h), with or without the addition of a non-steroidal anti-inflammatory ± amantadine (3–5 mg/kg PO q12 h) for the duration of the study.

### Blood Gas Analysis and Pain and Sedation Scoring

A peripheral venous sampling catheter was placed in the saphenous vein immediately post-operatively, prior to discontinuation of inhalant anesthesia, and was maintained for a maximum of 72 h, or until patency was lost. Previously published data from healthy dogs documents the relationship between blood gas variables from arterial and venous samples (12), but it is unknown that the same relationship holds true for arterial and venous blood samples in dogs with spinal cord injury undergoing anesthesia for imaging and surgical procedures. Therefore, in the first 14 patients, the arterial catheter that was placed early under anesthesia as part of standard of care was maintained in a similar fashion to the venous sampling catheter to confirm the relationship between arterial and venous blood gas values. After establishing appropriate correspondence, the remaining dogs in the study only had venous samples collected. If the sampling catheter lost patency prior to 72 h, all data collected prior was still used in analysis; if <3 blood samples remained for the study, venipuncture was performed for these remaining time points but if more than three samples remained, the remaining time points were nulled.

Blood gas measurements were performed on an in-house pHOx Ultra analyzer (Nova Biomedical, Waltham, MA). Base excess of blood (BE) was calculated as BE = (1 – 0.014 [Hb]) × ([${\text{HCO}}_{3}^{-}$] – 24 + (1.43[Hb] + 7.7) × (pH – 7.4)), according to the manufacturer's product manual. Partial pressure of arterial and venous oxygen concentrations (PaO_2_ and PvO_2_, respectively), blood pH, lactate, BE, and sedation and pain scores were assessed at 11 post-operative time points. Time 0 was defined as a timepoint after initiation of spontaneous ventilation (>4 breaths per minute) and disconnection of the anesthesia rebreathing circuit for at least 5 min, but before extubation. Time 1 was defined as a timepoint within the first 5 min after extubation. Blood samples were then drawn every 8 h after Time 1. Pain and sedation scales were assessed by intensive care unit (ICU) technicians using a visual analog scale (VAS) and simple descriptive scale (SDS), respectively (see Supplementary Material for an example blank scoring sheet). These technicians were provided training on the utilization of these scales prior to initiating the study.

### Definition of Subclinical Hypoventilation

Subclinical hypoventilation was defined as a PaCO_2_ ≥45 mmHg without clinical signs of hypoventilation (shallow breathing, open-mouthed “fish mouth” breathing, incomplete visible chest excursions).

### Statistics

Prior to enrollment, a preliminary power calculation was made using the following assumptions: The minimum clinically important difference we would like to be able to detect between groups would be a 5% increase in patients experiencing subclinical respiratory alterations associated with the ventral slot procedure compared to the hemilaminectomy. The estimated incidence of subclinical hypoventilation in the hemilaminectomy cases will be very low (≤0.5%). We wish to establish non-inferiority of cervical lesions relative to thoracolumbar lesions for preservation of ventilatory function with a statistical power of >80% at a significance level of 5%. We therefore estimated that 20 dogs in each of the two experimental groups would fulfill these assumptions.

Data are reported as mean ± SD or median (range) as appropriate based on the results of normality testing via the Kolmogorov–Smirnov method (13) (limiting form); continuous variables estimated PaCO_2_, pH, and BE met criteria for normality. Estimation of a linear relationship between arterial and venous samples was performed using a linear mixed-effects model with time of sample collection modeled as a random effect. Correlation between continuous variables was calculated as previously described for repeated-measures samples (14, 15). Relationship between estimated PaCO_2_, surgical group, and injury severity was assessed by multi-way ANOVA with time of sample collection considered as a grouping variable. The relationship between estimated PaCO_2_ and pain and sedation scores was evaluated by a linear mixed-effects model with time of sample collection modeled as a random effect. A Fisher's exact test was used to evaluate differences in proportions between groups. A *p* < 0.05 was considered statistically significant. Data analysis was performed using Matlab (Mathworks, Natick, NJ) version R2021a for Windows.

## Results

### Clinical Case Data

**Figure 1:**
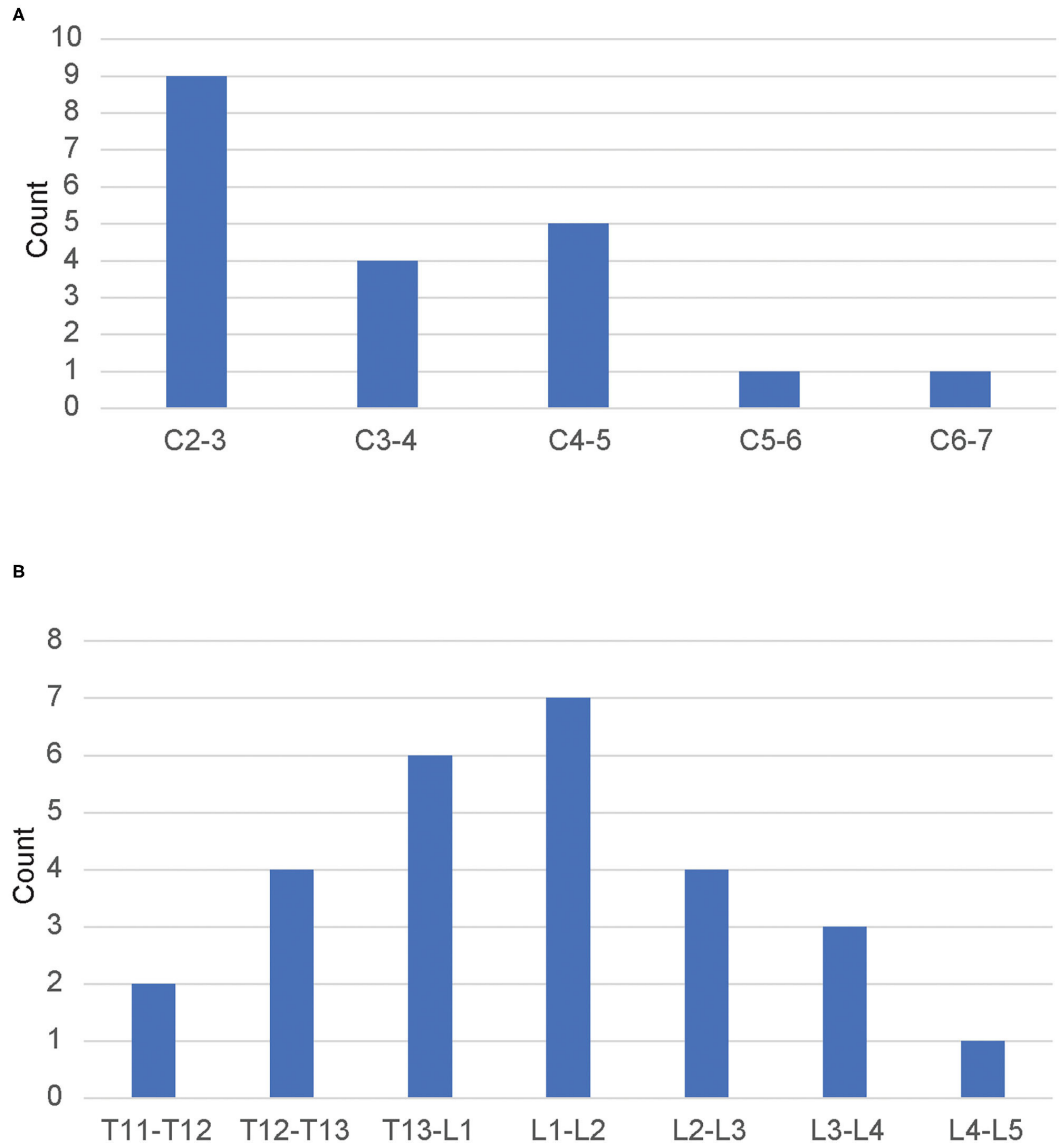
**(A)** Distribution of cervical lesions in dogs undergoing ventral slot decompression. *N* = 20. **(B)** Distribution of thoracolumbar lesions in dogs undergoing hemilaminectomy decompression. *N* = 20.

Of the 20 dogs with cervical lesions, all except one (Labrador Retriever) were small-breed, chondrodystrophic dogs, and 45% (9/20) were Dachshunds. The median age was 8 years (range 2.5–13 years). The median weight was 8.3 kg (3.4–42.6 kg). For this group, 35% (7/20) dogs were non-ambulatory at the time of surgery. Stratification by severity of spinal cord injury was as follows: pain only (7), ambulatory tetraparesis (6), non-ambulatory tetraparesis (5), and tetraplegia (2). One of the tetraplegic dogs had clinical hypoventilation diagnosed at presentation (pre-operatively) by blood gas and clinical signs. The most commonly affected intervertebral disc space was C2–3 (45%; 9/20), followed by C4–5 (25%; 5/20). Only two dogs had disc herniations caudal to C5 (Figure 1A). The median time under anesthesia was 5.0 h (range 4.0–6.5 h).

Of the 20 dogs with thoracolumbar lesions, all except one (Labrador Retriever) were small-breed, chondrodystrophic dogs, and 65% (13/20) were Dachshunds. The median age was 7.5 years (range 3–13 years). The median weight was 7.8 kg (range 4.8–27.8 kg). For this group, 70% (14/20) dogs were non-ambulatory at the time of surgery. The most commonly affected intervertebral disc space was L1–L2 (35%; 7/20), followed by T13–L1 (30%; 6/20). Four dogs (20%; 4/20) had lesions involving the lumbar intumescence (Figure 1B). The median time under anesthesia was 5.2 h (range 3.0–7.4 h).

### Post-operative Analgesia as a Function of Procedure

We investigated the use of post-operative analgesics in the two surgical groups. For dogs with cervical lesions undergoing ventral slot surgery, 90% (18/20) had continuous fentanyl administration post-operatively, 40% (8/20) had continuous ketamine administration, and 30% (6/20) had both. For dogs with thoracolumbar lesions undergoing hemilaminectomy, 95% (19/20) had continuous fentanyl administration, 15% (3/20) had continuous ketamine administration, and 15% (3/20) had both. There was no significant difference in the proportion of dogs receiving fentanyl (*p* = 1.0), ketamine (*p* = 0.74), or both between groups (*p* = 0.45).

### Correspondence of Arterial and Venous Samples

**Figure 2:**
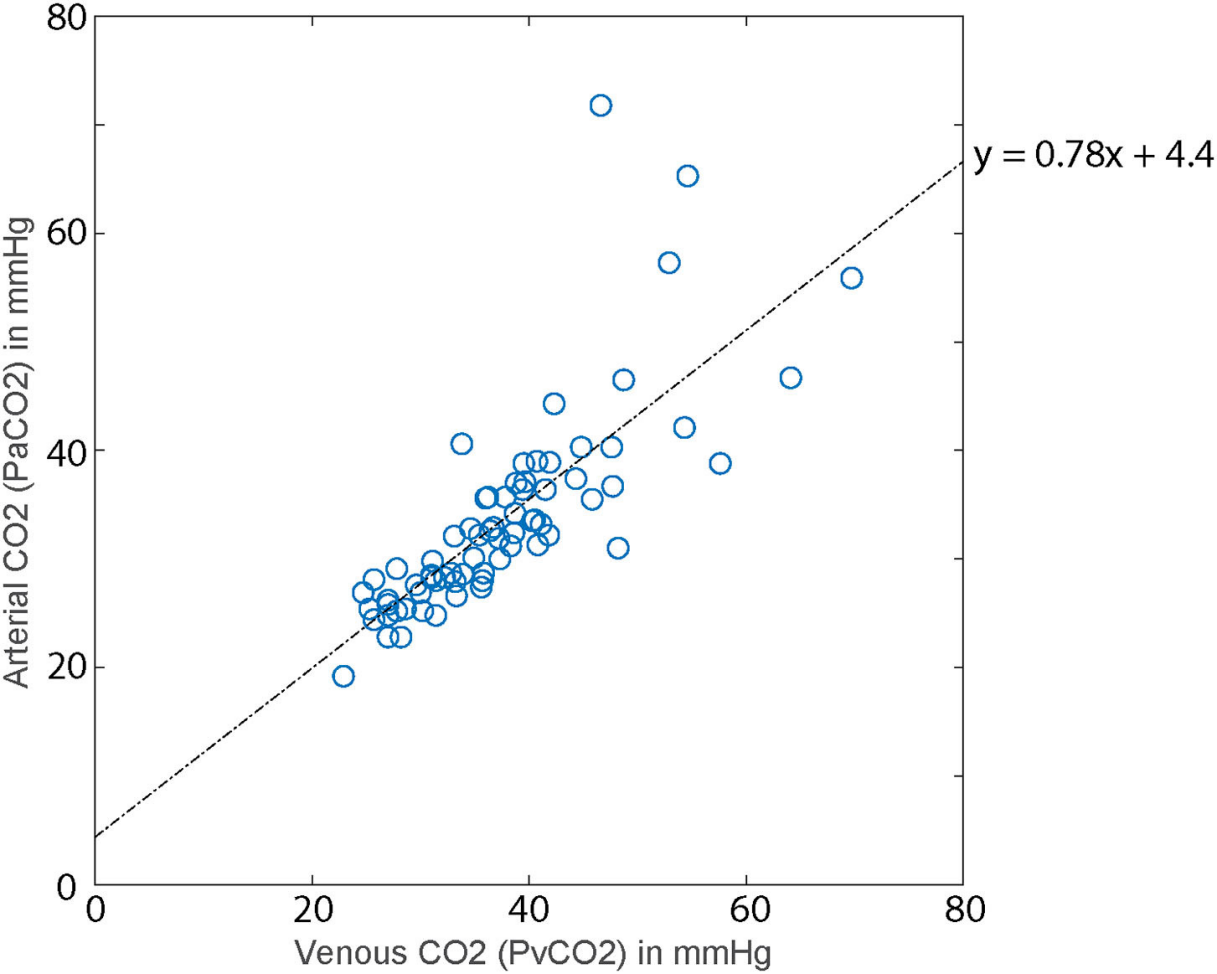
Linear relationship between venous CO_2_ and arterial CO_2_ for 71 paired samples. Dashed line is the regression line determined by linear mixed-effect modeling (*y* = 0.78*x* + 4.4).

There were 71 pairs of matched arterial (PaCO_2_) and venous (PvCO_2_) CO_2_ samples available for evaluation. These values were well-correlated (*R*_rm_ = 0.93; *p* < 10^−5^; Figure 2). A linear relationship between arterial and venous samples was approximated by PaCO_2_ = 0.78 (95% CI 0.63–0.93) × PvCO_2_ + 4.4 (95% CI −1.5–10.3). We used this formula to calculate estimated PaCO_2_ for all values for all patients at all timepoints.

### Documentation of Post-operative Ventilation

**Figure 3:**
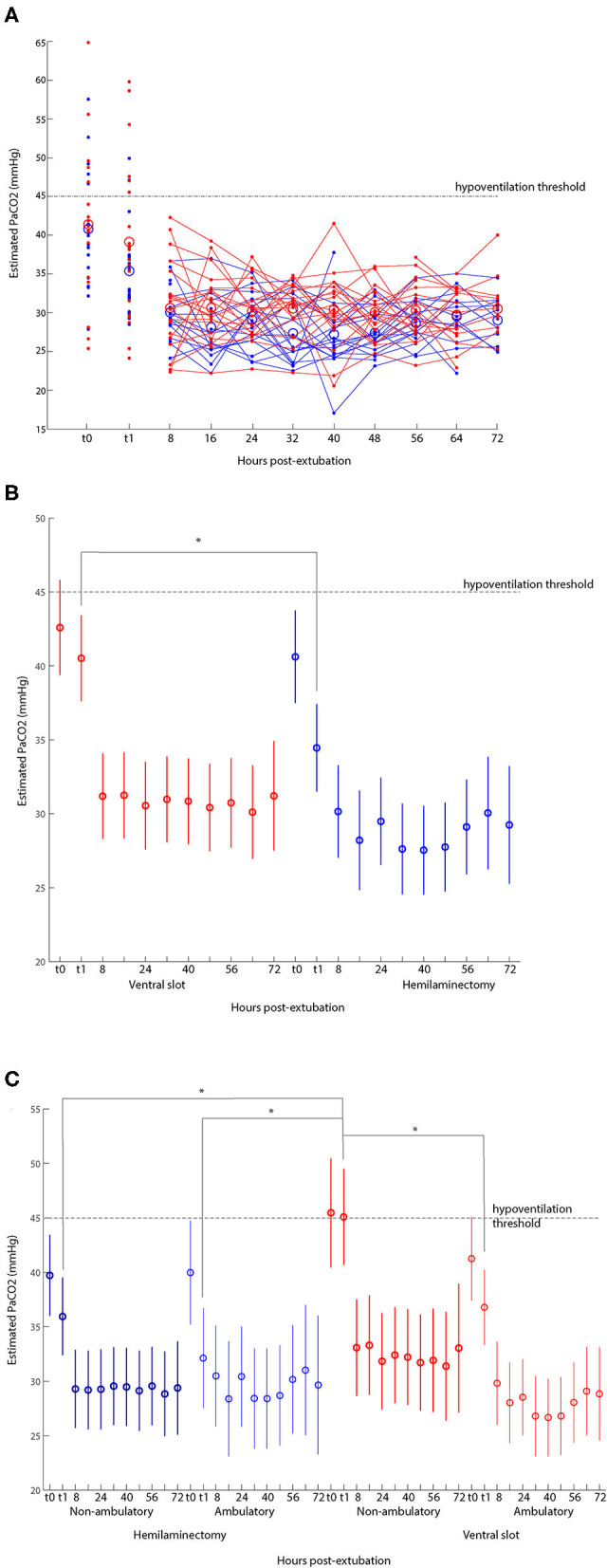
**(A)** Estimated PaCO_2_ as a function of time from extubation, stratified by surgical procedure/lesion location. Open circles represent means. Small dots are individual data points. Red data are from dogs undergoing ventral slot for a cervical lesion (*N* = 20) and blue data are from dogs undergoing hemilaminectomy for a thoracolumbar lesion (*N* = 20). Horizontal dotted line is estimated PaCO_2_ = 45 mmHg. Vertical dotted line is time of extubation. **(B)** Mean and standard error of estimated PaCO_2_ as a function of time and procedure determined by multi-way ANOVA. The asterisk represents significance at *p* < 0.05 for matched timepoints between hemilaminectomy and ventral slot groups. **(C)** Mean and standard error of estimated PaCO_2_ further stratified by severity of spinal cord injury, showing evidence of subclinical hypoventilation (defined as PaCO_2_ >45 mmHg) immediately before and after extubation in ventral slot patients with MFS <4 (non-ambulatory status) at presentation. Asterisks represent *p* < 0.05 for matched timepoints between groups determined by multi-way ANOVA.

Estimated PaCO_2_ for the two groups is plotted as a function of time in Figure 3A. Values >45 mmHg were common immediately before extubation and in the first 5 min following extubation for both groups. For patients undergoing ventral slot surgery, 25% (5/20) had estimated PaCO_2_ > 45 mmHg before extubation, and 30% (6/20) had estimated PaCO_2_ > 45 mmHg immediately after extubation. For patients undergoing hemilaminectomy, 30% (6/20) had estimated PaCO_2_ > 45 mmHg before extubation, and 10% (2/20) had PaCO_2_ > 45 mmHg immediately after extubation. These proportions were not statistically different (*p* = 1.0 and *p* = 0.24, respectively). No patient had estimated PaCO_2_ > 45 mmHg at any single time point later in recovery. The mean and range of estimated PaCO_2_ for each group at each time point is shown in Figure 3A. In a linear mixed-effects model, there was a significant effect of surgical group on estimated PaCO_2_ only in the immediate peri-extubation period (Figure 3B; *p* < 0.05). There was no relationship between the duration of anesthesia and PaCO_2_ at any timepoint (all *p* > 0.4).

### Relationship of Severity of Injury and Ventilation in Dogs With Cervical Lesions

We investigated ventilation as a function of severity of spinal cord injury (see section Materials and Methods). In the period immediately following extubation, there was a tendency for dogs with more severe injuries (those that were non-ambulatory pre-operatively) undergoing ventral slot surgery to have higher estimated PaCO_2_ (Figure 3C; *p* < 0.005).

### Relationship Between Other Markers of Acid-Base Status and Ventilation

**Figure 4:**
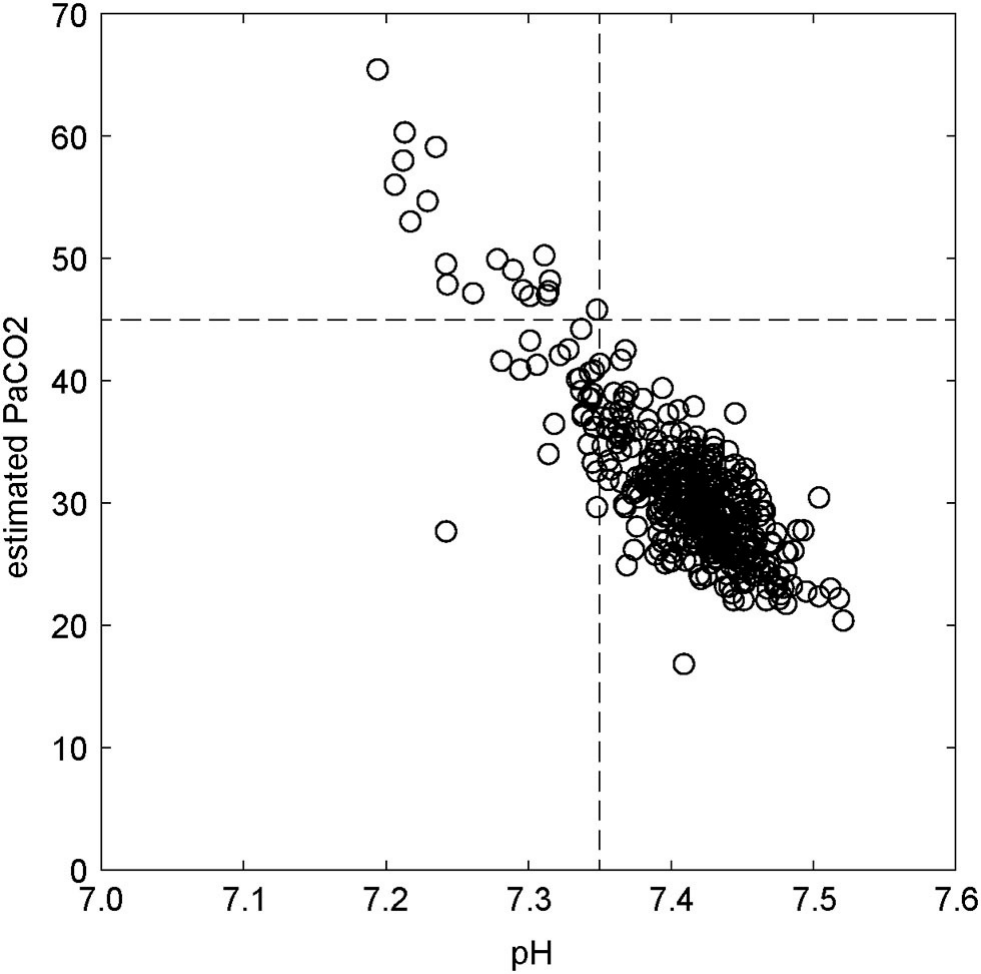
Relationship between estimated PaCO_2_ and pH. Horizontal dotted line is estimated PaCO_2_ = 45 mmHg. Vertical dotted line is pH = 7.35.

There was an inverse linear relationship between pH and estimated PaCO_2_, demonstrated in Figure 4, such that increasing estimated PaCO_2_ values were associated with lower pH values (*R*_rm_ = −0.62; *p* < 10^−5^). The majority of estimated PaCO_2_ > 45 mmHg occurred in conditions of acidosis (pH < 7.35). The maximum calculated BE across all patients and timepoints was 5.0 mmol/L (median −3.4 mmol/L, normal reference range for our analyzer −5 to 5 mmol/L). Four patients (two ventral slot and two hemilaminectomies) exhibited BE < -5 mmol/L under conditions of acidosis for one or two timepoints, but the patients were not hypercapnic at those times. Four patients exhibited acidosis (pH < 7.35) associated with both elevated PaCO_2_ [48.8, 47.5, 47.6 (ventral slot) and 56.1 (hemilaminectomy) mmHg] and simultaneous elevated lactate (2.8, 3.2, 2.8, and 3.1 mmol/L, respectively) at a single timepoint each. Further comparisons and analysis of acid-base status were not performed, since they are outside the scope of this study.

### Relationship Between VAS, SDS, and Ventilation

**Figure 5:**
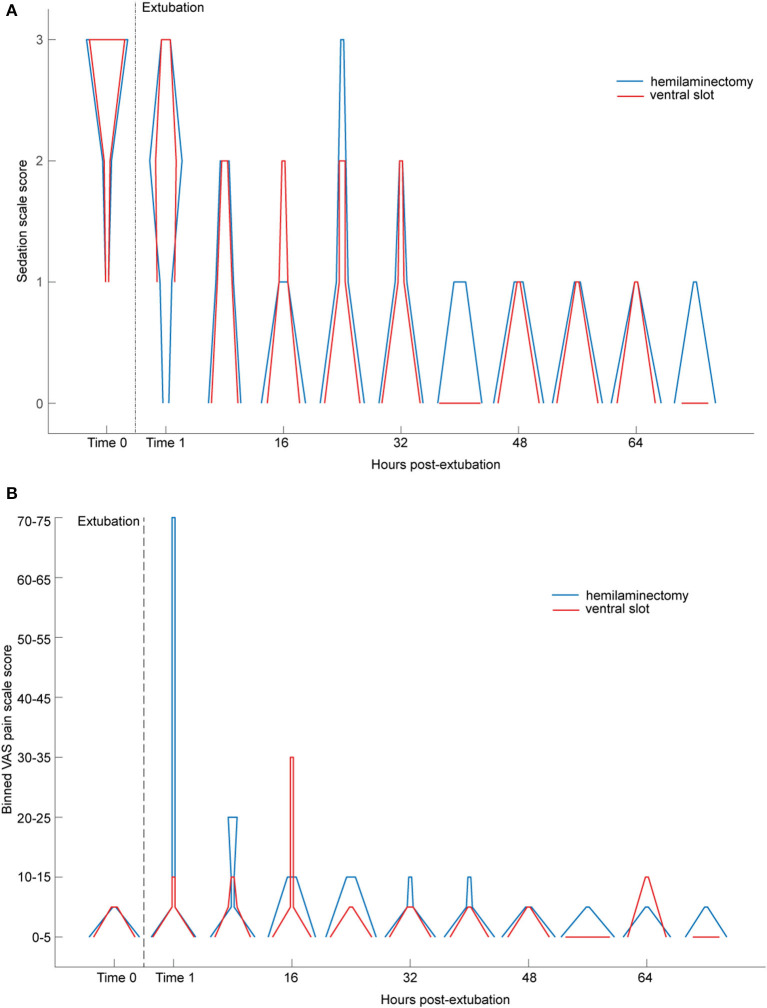
**(A)** Sedation scale score as a function of time from extubation, stratified by surgical procedure/lesion location. The width of the horizontal line at each sedation score level (y-axis) represents the number of dogs for which that score was recorded (out of 20 possible) at that time point; wider horizontal width means the score was recorded more frequently. Red lines are for dogs undergoing hemilaminectomy; blue lines are for dogs undergoing ventral slot. Dotted vertical line is time of extubation. **(B)** VAS pain score as a function of time from extubation, stratified by surgical procedure/lesion location. Open circles represent means. Small dots are individual data points. Red data are from dogs undergoing ventral slot for a cervical lesion (*N* = 20), and blue data are from dogs undergoing hemilaminectomy for a thoracolumbar lesion (*N* = 20). Horizontal dotted line is estimated PaCO_2_ = 45 mmHg. Vertical dotted line is time of extubation. **(C)** Box and whiskers plot of one-way ANOVA of the effect of sedation scale score on estimated PaCO_2_. On each box, the central mark is the median, and the edges of the box are the 25th (*q*_1_) and 75th (*q*_3_) percentiles. The whiskers extend to the most extreme data points that are not considered outliers. The outliers are plotted individually using the “+” symbol. The extremes of the whiskers correspond to *q*_3_ + 1.5 × (*q*_3_ – *q*_1_) and *q*_1_ – 1.5 × (*q*_3_ – *q*_1_).

**Figure 6:**
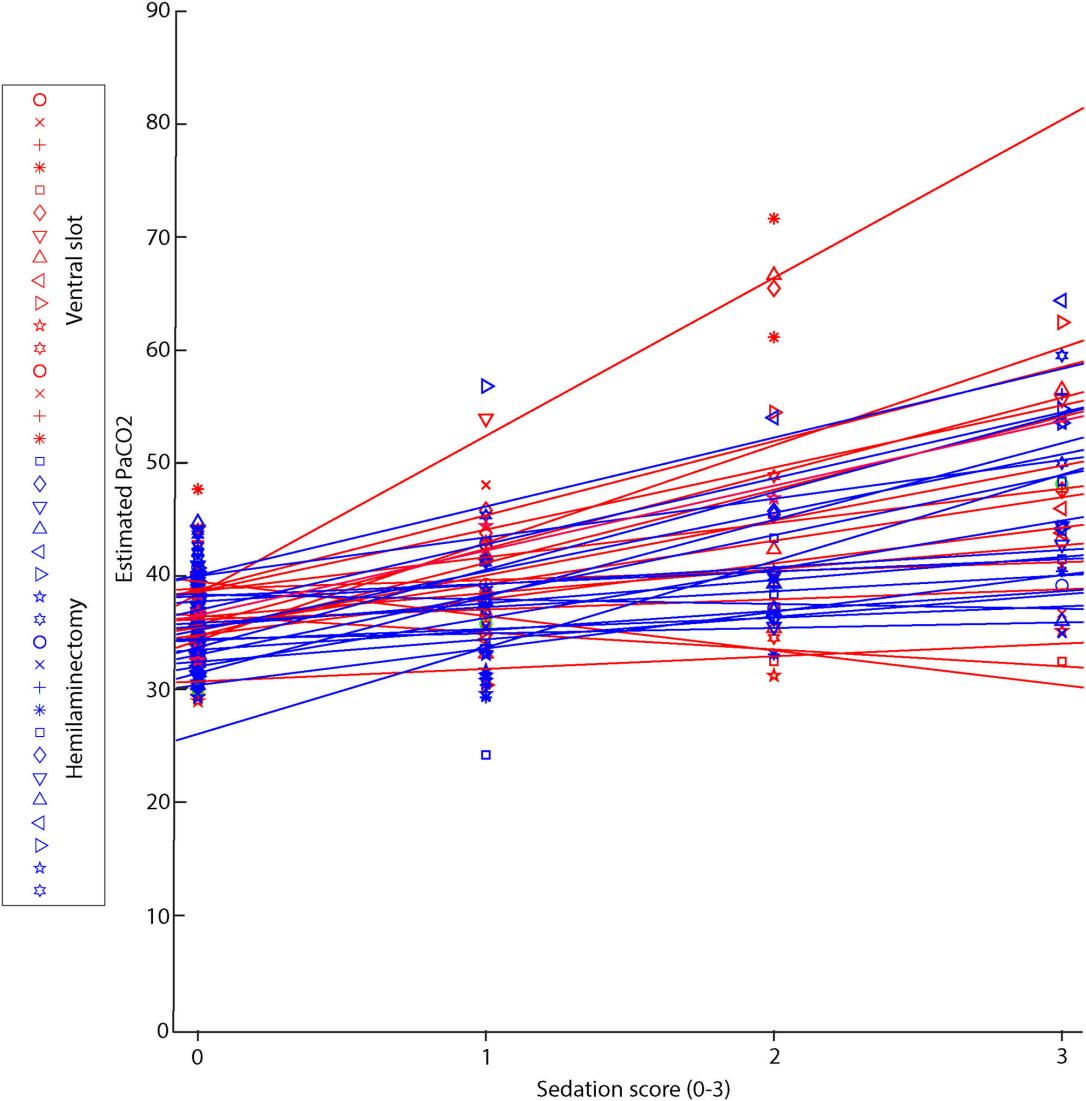
Individual linear estimations of the relationship between estimated PaCO_2_ and sedation score (SDS) for all patients. Those undergoing ventral slot are represented by red symbols and lines (*N* = 20) and those undergoing hemilaminectomy are represented by blue symbols and lines (*N* = 20). Higher sedation scores indicate a greater degree of sedation.

Overall, pain scores assessed by the VAS and sedation scores assessed by the SDS were low, except for one outlier in the hemilaminectomy group. Simple descriptive scale scores decreased with increasing time from extubation (Figure 5A), independent of lesion location. Visual analog scale scores were highest in the first 16 h after extubation (Figure 5B). Figure 6 shows the relationship between estimated PaCO_2_ and SDS for each individual, which exhibited a moderate positive correlation (*R*_rm_ = 0.66; *p* < 10^−5^). There was no linear relationship evident between estimated PaCO_2_ and the VAS (*p* = 0.4). Further, we did not find relationship between the particular post-operative analgesics selected (fentanyl, ketamine, or combination) and SDS (*p* = 0.64, *p* = 0.82, *p* = 0.74, respectively)_._

## Discussion

The risk of hypoventilation may have insidious consequences for a healing spinal cord. It is known that PaO_2_, PaCO_2_, and systemic blood pressure can affect spinal cord tissue oxygen levels (16–18). This is explained by the fact that autoregulation of spinal cord blood flow is compromised in the face of hypotension, hypercarbia, and hypoxia (16). Olby (19) stressed that the loss of spinal cord autoregulation along with concurrent systemic hypotension can negatively impact overall outcome after spinal cord injury. Some patients that exhibit clinical hypoventilation are easy to identify, but the clinical signs of hypoventilation may be subtle and differ from other forms of respiratory distress, so such patients may go unrecognized until their condition is severe. The incidence of subclinical hypoventilation in veterinary patients undergoing decompressive surgery for spinal cord injury has not been previously explored.

This study did not identify a prolonged increased risk of subclinical hypoventilation for dogs undergoing decompressive surgery for cervical IVDH compared to dogs undergoing decompressive surgery for thoracolumbar IVDH. Neither group showed evidence for subclinical hypoventilation after the peri-extubation period during anesthetic recovery. This is consistent with previous reports indicating that clinical hypoventilation in dogs undergoing these surgeries is infrequent (1, 3–5, 8). In these prior studies, the exact times at which post-operative recognition of hypoventilation occurred are not reported. Our study indicates that subclinical hypoventilation may occur without being recognized in patients that are not assessed frequently and specifically for hypercapnia in early recovery.

Because multiple anesthetic events have been previously identified as a risk factor for respiratory complications after spinal surgery that may be confounded with cervical lesion location (4), we deliberately restricted our sample population to dogs that experienced just one anesthetic event for both imaging and surgery. Further, we did not evaluate other surgical approaches to the cervical region outside of the ventral slot surgery. Historically, reports of respiratory complications following cervical surgery have included dorsal approaches and atlanto-axial stabilization (1), which may carry higher risk.

In this population of dogs, a correlation between the severity of clinical neurologic dysfunction and the development of subclinical hypoventilation post-operatively was found only in the immediate peri-extubation period. Specifically, injury causing clinical signs of severe cervical spinal cord dysfunction was associated with subclinical hypoventilation assessed within 5 min of extubation.

For individuals in both groups, subclinical hypoventilation occurred early in the post-operative period, during recovery from anesthesia, while the patients were still being monitored for respiratory status every 5 min and did not occur outside of this window. Ventilatory parameters were correlated with SDS, with both SDS and PaCO_2_ being highest in the immediate peri-extubation period. This finding suggests that the subclinical hypoventilation noted in this population may be a manifestation of anesthesia-related sedation; however, the design of this study does not allow us to determine if there is a causal relationship between SDS and ventilation status. Alternatively, early and transient post-operative subclinical hypoventilation may be related to atelectasis that develops intraoperatively. Though neither of these explanations represents neurological impairment of ventilation, they are still potential risk factors for hypercapnia in early recovery and deserve further investigation. Another possible factor related to ventilatory compromise in early post-operative recovery might be body condition, which was not assessed in our study.

Elevated PaCO_2_ values were correlated with decreased pH, consistent with a respiratory acidosis in those patients affected. Comparative changes in BE suggestive of mixed acidosis were rare and deemed to have minimal significance in this population.

One limitation of our study is that timepoints between 5 min after extubation and 8 h after extubation were not assessed, so the duration of subclinical hypoventilation in affected patients could not be established with precision. In our study population, all but one dog had normal ventilation status pre-operatively, so we cannot comment on post-operative ventilatory stability in patients that exhibit pre-operative clinical hypoventilation. Nevertheless, the one dog exhibiting clinical hypoventilation on initial presentation (tetraplegia; EtCO_2_ > 50 mmHg; PvCO_2_ > 50 mmHg; short, shallow breaths, and “fish-mouth” breathing) did not exhibit either clinical or subclinical hypoventilation post-spinal cord decompression. Larger studies, especially those including more severely injured patients, would be warranted to determine the clinical significance, if any, of subclinical hypoventilation. Questions of clinical interest that could be addressed by such studies include the relationship between specific sites of cervical injury and subclinical hypoventilation, and the possibility of association between subclinical hypoventilation and outcome.

## Conclusions

Subclinical hypoventilation was seen in post-operative ventral slot patients with severe cervical spinal cord dysfunction but was transient and may have been associated with sedation or atelectasis during early anesthetic recovery. The results of this study indicate that dogs undergoing routine ventral slot or hemilaminectomy procedures that do not exhibit subclinical hypoventilation in early recovery are unlikely to develop hypoventilation later in recovery, and that some dogs show a transient subclinical hypoventilation in early recovery that can be self-limiting. However, further studies are needed to assess if clinical hypoventilation on presentation is associated with likelihood of persistent clinical hypoventilation after spinal decompressive surgery, as well as to determine the exact duration and cause of subclinical hypoventilation in affected patients following anesthetic recovery.

## Data Availability Statement

The original contributions presented in the study are included in the article/Supplementary Material, further inquiries can be directed to the corresponding author/s.

## Ethics Statement

The animal study was reviewed and approved by Texas A&M University Institutional Animal Care and Use Committee. Written informed consent was obtained from the owners for the participation of their animals in this study.

## Author Contributions

MA was responsible for subject enrollment, data collection, and collation. MA and EB contributed to data analysis and the original manuscript draft. All authors contributed to study design and manuscript development.

## Conflict of Interest

The authors declare that the research was conducted in the absence of any commercial or financial relationships that could be construed as a potential conflict of interest.

## Publisher's Note

All claims expressed in this article are solely those of the authors and do not necessarily represent those of their affiliated organizations, or those of the publisher, the editors and the reviewers. Any product that may be evaluated in this article, or claim that may be made by its manufacturer, is not guaranteed or endorsed by the publisher.
